# Contagion of Consumption

**Published:** 1880-09

**Authors:** James T. Whittaker


FROM A PAPER ON THE CONTAGION OF CONSUMPTION. By JAMES T. WHITTAKER, M.D.

The whole question of the contagion of consumption, *i. e.* of the specificity of the tuberculous virus, hinges upon its inoculability, and of this capability there is now scarcely room for doubt. Harnsell (Graefe's *Archiv.*, 25, part iv, 1879) mentions cases of tuberculosis iridis, as reported by Perls, Manfredi, Koester, Leber, Samelsohn, Sattler and Angelucci, and adds three cases from his own (Göttingen) clinic. He thus establishes the fact of infection of the iris. After mentioning the experiments of others in direct and indirect inoculation of the eye with tuberculous matter, the author then proceeds to detail his own. He found that the insertion of tuberculous matter into the anterior chamber of the eye invariably inoculated the iris. The cornea and conjunctiva, moreover, could be inoculated directly, and in all cases the tuberculous matter inserted "disappeared by the third day, and after from fifteen to twenty-three days of incubation tuberculous collections showed themselves."

The fact that these collections or masses were tubercular was proven after the method of the chemists in recognition of the action of a poison, that is, by insertion into the bodies of other animals. Particles or portions were put into the abdominal cavities of dogs and guinea-pigs. The dogs died of suppurative peritonitis. The guinea-pigs were kept under observation for three months and then killed, when "all the internal organs and the skin were found, without exception, to be filled with deposits of miliary tubercles." Cohnheim tried in vain to excite tubercles in the iris "by introducing into the anterior chamber portions of non-tuberculous animal tissues, of the most varied kind," and Harnsell failed to inoculate tuberculosis with fresh trachomatous matter. "On the other hand, the tuberculous matter used, when introduced into the peritoneal cavity, excited, in turn, general tuberculosis of all the organs."

"*So in Tuberculosis*," as Cohnheim concludes, "*everything depends upon the virus. We discover at all points the closest analogies between tuberculosis and syphilis. Both require, above all things, infection, transmissibility of the disease from person to person.*"

This comparison between tuberculosis and syphilis is exceedingly happy. The conduct of no other infectious disease so closely resembles that of tuberculosis, or so completely clears up the perplexities which beset the disease. To compare tuberculosis with small-pox, for instance, would be fatal to our understanding of either, while tuberculosis and syphilis present so many analogies as to have even led some pathologists to regard one as a form or a product of the other, a conception which is, of course, radically wrong.

Tuberculosis, like syphilis, depends then upon a specific virus which must reach a mucous membrane or a broken surface, to be absorbed and induce the disease. Laennec was convinced that he had inoculated himself once with tuberculosis, just as many an unfortunate practitioner has since inoculated himself with syphilis, by a wound from a saw in making a post-mortem examination upon a phthisical patient. But more fortunate than they, he succeeded in destroying the tuberculous nodule at the start with the butter of antimony.

Syphilis, for the most part, reaches the body through the organs of generation, while tuberculosis is breathed, for the most part, into the lungs, or is swallowed with food, as with milk, the most frequent cause of tuberculosis in childhood.

The first symptoms of each affection are local; in syphilis, at the genital organs, in tuberculosis, at the lungs or in the intestinal canal. From the point of absorption the disease next invades the lymph glands in the nearest vicinity; in syphilis, the glands in the groin, in tuberculosis, the bronchial and mesenteric glands. Passing these glands, or being absorbed into the blood, both diseases become general.

Both diseases may be transmitted by heredity, and both diseases, thus transmitted, may lie latent for a time, for a longer time in tuberculosis, to develop at a later period. During the latent stages, both diseases impair the processes of nutrition, and deform the development of the body. The victim of latent syphilis has notched teeth, falling hair, derangements of digestion, etc. The victim of tuberculosis shows the aspect of scrofula, or has the sunken, elongated chest, ossified ribs, the thorax paralytica; he has also clubbed fingers, and the other well known signs which constitute the *phthisical habitus*. This habitus is therefore an effect and not an inviting cause of the disease. An individual thus affected is said to have the tuberculous diathesis, just as an individual once syphilized has the syphilitic diathesis. Either disease may manifest itself in its well known symptoms at any time. But latent or manifest, the disease is present, just the same, all the time.

There is, then, no such thing as a predisposition to either disease. Either a man has syphilis or he has it not. Either a man has tuberculosis or he has it not. One man is not more predisposed to either disease than another. Syphilis affects one individual more than another, because its virus finds a better lodgment upon his mucous membranes. Tuberculosis finds also, fortuitously, a better nidus in one case than another. The virus of tuberculosis is lodged in one case and not coughed up, just as in syphilis the virus is secreted and not washed off.

Both diseases may disappear from the body entirely, and a perfect cure may result, but it is impossible to state when such complete eradication has taken place. In syphilis the capability of reinoculation furnishes the only definite information in this regard, a method of trial not so likely to be undertaken in tuberculosis. As a rule, however, neither disease does disappear from the body entirely. What Fournier said of syphilis is true also of tuberculosis, viz., that the diathesis is a period of health interrupted by explosions of the disease. Cazenave said long ago that one does not recover from the syphilitic diathesis, but lives with it as with the lymphatic temperament, and an older writer observed that syphilis strikes with its victims "a truce oftener than a peace."

Both diseases may, and for the most part do leave in the body centers of future infection. From any chancre, plaque, gumma, or other deposit of syphilis, reabsorption may take place at any time, and reinfection with syphilis, or, better, reappearance of external signs. So, from any caseous nodule wherein the tuberculous virus is locked up in temporary innocence, absorption may take place under favoring circumstances and a new outbreak of tuberculous symptoms appear, the quantity of virus thus set free, determining to great extent, perhaps, the virulence of the symptoms. While the virus is thus locked up the disease is latent, when set free, it is manifest.

While it is true, therefore, of both diseases that they may be inherited, that is, that both syphilis and tuberculosis may affect the ova and spermatozoids as well as every other organ and tissue of the body, it is also true of both diseases that they are in the vast majority of cases not inherited, but acquired. A thorough sifting of the cases will show this statement to be as notoriously true of tuberculosis as of syphilis. So soon as the inoculability of tuberculosis is established, the fact is also established that the disease is acquired oftener than inherited.

With the general recognition of these views, we shall cease to hear of bad air and bad sanitation as direct factors in the disease. The writer of this article once went so far as to develop tuberculosis from depressing mental emotions. Bad air, food or drink, are productive of tuberculosis only when they contain the virus of the disease. In other respects they are no worse for tuberculosis than for any other disease. Drinking water contaminated with sewage does not produce typhoid fever unless the sewage contain the typhoid germ. So, contaminated air is productive of tuberculosis only when a cause of its contamination is tuberculous virus. Drs. Cotton and Edwards, of the Brompton Hospital for Consumptives, object to the contagiousness of consumption on the ground that but one nurse and one servant died of phthisis in that institution in a period of twenty-one years. Dr. Cotton went so far as to say that "a residence in the consumptive hospital and long continued working in its wards is a very good way, indeed, *not to catch the disease.*" It must be remembered, however, that few institutions were in such perfect sanitation, especially as regards ventilation, as Brompton Hospital. Anyhow, the statement does not count for much less than to show how close an association is necessary to contract the disease. For the same observation has been made with reference to typoid fever, an infectious disease beyond a doubt. Liebermeister states that up to the year 1865 he had never seee in the hospitals which he visited (Greifswald, Berlin, Tübingen) "a single hospital patient, physician or nurse, attacked with typhoid fever, although such cases are placed in the general wards." And the same author quotes from Murchison to the effect that "during a period of fourteen and a half years in the London Fever Hospital, 2,506 patients were treated with typhoid fever, and during that time only eight cases originated in the hospital."

The specificity of the tuberculosis virus is determined in a higher school, and by means more in accord with the principles of science than clinical observation. And the recognition of it clears the field for prophylaxis and opens up a new and more promising outlook for the therapy of the disease.

---

Dr. Stuart Eldridge, Gen. Hospital, Yokohama, Japan, calls the attention of the profession to the great advantages of Asbestos roofing felt as a material for plastic splints and other molded apparatus. In a brief statement he gives the qualities in which it excels those now in vogue. 1. It is rendered perfectly soft and flexible by brief immersion in water of a temperature easily borne by the hand. 2. It retains its plasticity long enough to allow of careful adaptation, while its stiffness is instantly restored by a dash of cold water. 3. While soft it does not change dimensions, so often the case with gutta percha. 4. It remains unchanged after indefinite exposure to the heat and moisture of the body, nor is it affected by any of the ordinary lotions applied in cases of wound or fracture. 5. It is perfectly antiseptic on account of the coal tar with which it is saturated, a quality which would of itself commend its use in compound fracture. 6. It is so cheap that its cost is hardly worth mentioning even in large institutions.—*N. Y. Med. Record.*

